# Active Fungal Communities in Asymptomatic *Eucalyptus grandis* Stems Differ between a Susceptible and Resistant Clone

**DOI:** 10.3390/microorganisms7100375

**Published:** 2019-09-20

**Authors:** Mandy Messal, Bernard Slippers, Sanushka Naidoo, Oliver Bezuidt, Martin Kemler

**Affiliations:** 1 Department of Biochemistry, Genetics and Microbiology, Forestry and Agricultural Biotechnology Institute, University of Pretoria, P. Bag X20, Hatfield 0028, Pretoria 0002, South Africa; bernard.slippers@fabi.up.ac.za (B.S.); sanushka.naidoo@fabi.up.ac.za (S.N.); 2 Department of Biochemistry, Genetics and Microbiology, Centre for Microbial Ecology and Genomics, University of Pretoria, P. Bag X20, Hatfield 0028, Pretoria 0002, South Africa; bezuidt@gmail.com; 3 AG Geobotanik, Ruhr-Universität Bochum, Gebäude ND 1/150, Universitätsstraße 150, 44780 Bochum, Germany

**Keywords:** asymptomatic plant infection, plant–fungus interaction, plant–microbe interaction, secreted proteins, metatranscriptomics, CAZymes, pathogen–host interaction

## Abstract

Fungi represent a common and diverse part of the microbial communities that associate with plants. They also commonly colonise various plant parts asymptomatically. The molecular mechanisms of these interactions are, however, poorly understood. In this study we use transcriptomic data from *Eucalyptus grandis,* to demonstrate that RNA-seq data are a neglected source of information to study fungal–host interactions, by exploring the fungal transcripts they inevitably contain. We identified fungal transcripts from *E. grandis* data based on their sequence dissimilarity to the *E. grandis* genome and predicted biological functions. Taxonomic classifications identified, amongst other fungi, many well-known pathogenic fungal taxa in the asymptomatic tissue of *E. grandis*. The comparison of a clone of *E. grandis* resistant to *Chrysoporthe austroafricana* with a susceptible clone revealed a significant difference in the number of fungal transcripts, while the number of fungal taxa was not substantially affected. Classifications of transcripts based on their respective biological functions showed that the fungal communities of the two *E. grandis* clones associate with fundamental biological processes, with some notable differences. To shield the greater host defence machinery in the resistant *E. grandis* clone, fungi produce more secondary metabolites, whereas the environment for fungi associated with the susceptible *E. grandis* clone is more conducive for building fungal cellular structures and biomass growth. Secreted proteins included carbohydrate active enzymes that potentially are involved in fungal–plant and fungal–microbe interactions. While plant transcriptome datasets cannot replace the need for designed experiments to probe plant–microbe interactions at a molecular level, they clearly hold potential to add to the understanding of the diversity of plant–microbe interactions.

## 1. Introduction

Fungal–plant interactions are essential components in plant physiology, ecology and evolution. Fungal pathogens for instance can have a devastating impact on agriculture and forestry [1], but are also known to alter entire natural ecosystems through large-scale outbreaks [2]. Conversely, mycorrhizal interactions contribute significantly to the nutrition and water acquisition in approximately 85% of all plant species [3]. Both types of interactions are known to induce substantial phenotypic changes in the host plant. In the case of pathogens, this can be seen in abnormal growth, whereas mycorrhizal interactions lead to the formation of specific morphological root structures (e.g., haustoria or the Hartig net).

A more cryptic plant–fungal interaction involves the growth of fungal endophytes within plants or as epiphytes on plant surfaces, without causing any visible symptoms in the host [4,5,6]. Due to this hidden lifestyle, both endophytic and epiphytic fungi have, until recently, received only limited attention. But a growing number of studies shows that endophytic and epiphytic fungi can provide vital functions, such as plant pathogen inhibition [7]. Recent taxonomic surveys using next generation sequencing (NGS) of PCR amplicons have identified an enormous diversity of endophytic and epiphytic fungi associated with plants [8,9,10], implying that the importance of these interactions is underestimated. Especially, the impact of the unaccounted microbial gene repertoire and the accompanying metabolic potential provided by the microorganisms to their host are largely unknown. This arises from the difficulties of investigating the functional aspect of fungal plant interactions in vivo and the fact that physiologically active fungal molecules represent only a small fraction of the total molecular pool.

At a molecular level, antagonistic and beneficial plant–fungus interactions are often characterised by acutely fine-tuned interactions between both partners. Prior to the formation of specific morphological structures, the interactions involve the molecular recognition and subsequent respective reactions of the interacting partners. In both beneficial and pathogenic interactions, the molecules secreted by both partners facilitate the development of the association between the organisms [11].

Pathogen recognition by plants is achieved using two categories of perceptions [12]. Pathogen-associated molecular patterns (PAMPs), such as chitin that are common to many fungal pathogens, are recognised by extracellular plant pattern recognition receptors (PRRs) of plants and lead to PAMP-triggered immunity [12]. Virulence factors (effectors) produced by more specialised pathogens are injected into the plant cell and are potentially recognised by intracellular receptors of the plant to stimulate effector-triggered immunity [13]. Effectors are also important in ectomycorrhizal interactions and help to repress plant defence responses during infection and most likely in later stages of the mutualistic symbiosis [14,15]. After successful establishment of the interaction between the host and the infecting fungus, a bidirectional flux of nutrients is established at the interaction zone [16].

Fungi involved in asymptomatic plant associations are often closely related to disease-causing genotypes or mycorrhizal fungi [17]. It must be assumed that similar molecular mechanisms are applied in all of these interactions, but little is currently known of either the effectors or physiological responses of asymptomatic associations. Metagenomic studies, using shotgun sequence technology, explore microbial taxonomy in environmental DNA samples and can predict potential microbial functions [18]. An approach rarely explored to investigate fungal–plant interactions is the use of plant RNA-seq data from healthy plants and to filter for fungal transcripts. The use of high-throughput sequencing should provide suitable sequence datasets due to the high sequencing depths attainable. RNA-seq data provide information about gene expression, and although not the primarily purpose, can provide taxonomic information, which contribute to the understanding of the diversity and function of fungi involved in plant interactions. Previous studies already recovered fungal transcripts as a by-product during the analysis of plant transcriptomes [19] and annotated them with some general biological function [20]. However, the use of specific databases for plant–microbe interactions additionally facilitates a more detailed analyses of biological function for this fungal transcriptome data.

In this study we re-analyse transcriptomic data of stem tissue from a study that investigated the *Eucalyptus grandis*–*Chrysoporthe austroafricana* interaction in two different *E. grandis* clone lineages that are partially resistant and highly susceptible to *C. austroafricana*, the causal agent of stem canker [21]. We used the RNA-seq data to address three questions: (1) Is it possible to recover fungal transcripts from the plant transcriptome data in a sufficient amount to describe the fungal communities within the two *E. grandis* clones? (2) If so, what is the taxonomic composition of the fungal transcripts identified? (3) What are the potential functions of the fungal transcripts and are these putative functions related to known plant–fungal interactions?

## 2. Materials and Methods

Paired-end mRNA reads from a published *E. grandis* data set [21] were obtained to study transcripts of associated fungi. In this previous study, mRNA was extracted from stem tissue of the *E. grandis* clones TAG5 and ZG14 grown in a greenhouse trial set up to understand the *E. grandis* defence response against *C. austroafricana*. In the interaction with *C. austroafricana*, the *E. grandis* clone TAG5 is moderately resistant while ZG14 is highly susceptible. For the purpose of our study, we analysed data from mock-inoculated (control), as well as from *C. austroafricana* infected stems, which were collected from three individuals for each clone per treatment at a single time point [21].

### 2.1. Taxonomic and Functional Classifications of Transcripts

Complete mRNA data were mapped individually for each sample against the *E. grandis* genome V2 [22] using TopHat 2.1.1 [23]. All reads that did not map against the *E. grandis* genome were subsequently de novo assembled using Trinity V2.4.0 [24], which included trimming of reads using Trimmomatic [25] when the Phred quality score fell below 30. Trinity uses de Bruijn graph partitioning to assemble small reads into larger contigs for annotation [26]. To search for the most similar proteins and source organisms, all contigs were aligned against the NCBI non-redundant (nr) protein database (V83) using blastx [27] with an e-value threshold of 1 × 10^−5^. Trinity and blast output files were used directly as input for MEGAN 6.12.3 [28]. MEGAN computes and explores the taxonomic content based on the NCBI taxonomy and assigns each contig to a taxon node using a lowest common ancestor algorithm. Sequences assigned to the node “Fungi” were extracted in MEGAN and only these were used in subsequent analyses. The control and infected data for both clones were used to compare the number of fungal transcripts between the different conditions.

Control and infected data sets were separated, and functional analyses were only conducted on control data. Potentially functional genes of the six TAG5 and ZG14 control data sets of the extracted fungal transcriptome data were analysed individually using the Eukaryotic Non-Model Transcriptome Annotation Pipeline (EnTAP) 0.8.2 [29] and Blast2GO^®^ 5.2 [30] using nucleotide sequences. Frame selection implemented in GenMarkS-T 5.1 [31], was used to predict the most likely Open Reading Frame (ORF) and identify protein-coding regions in the RNA transcripts. Local InterProScan 5.24 [32] and NCBI-BLAST 2.7.0 blastx, using an e-value of 0.001, were used to annotate the transcripts based on fungal nucleotide data. Blast2GO was then used to predict gene ontology (GO) in the categories ‘biological processes’, ‘molecular function’ and ‘cellular component’.

Functional annotation using eggNOG-Mapper 0.12.7 [33] under default settings based on the eggNOG 4.5 orthology database [34] was performed on combined TAG5 and combined ZG14 protein sequences from controls, respectively. The database was adjusted to the taxonomic scope fuNOG to establish likely functional classifications against the EuKaryotic Orthologous Groups (KOG) [35]. KOG identified proteins were grouped based on the corresponding KOG classes.

### 2.2. Pathogen–Host Interaction Prediction

Potential roles of transcripts in fungus-plant interaction were determined using blastp implemented in DIAMOND 0.8.31 [36] using an e-value of 0.0001 with protein sequences translated in EnTAP against the pathogen–host interaction database PHI-base 4.5 [37]. Hits were grouped according to PHI-base knock-out phenotypes.

### 2.3. Prediction of Secreted Proteins

To identify secreted proteins that potentially mediate microbial interaction with their hosts and with other microbial community members, we filtered the fungal transcripts using several databases. Protein sequences, translated in EnTAP, were used for prediction of secretory proteins using a combination of three tools: SignalP 3.0 [38], TargetP 1.1 [39] and TMHMM 2.0 [40]. Protein sequences provided as input to SignalP with both, a D score and signal peptide probability ≥0.5 were classified as classically secreted and those with scores <0.5 as non-secreted. Classically secreted proteins were provided as input to TargetP to predict mitochondrial proteins. Sequences predicted as mitochondrial proteins were not considered and removed from the data set. The remaining sequences (i.e., those not predicted as mitochondrial proteins) were provided as input to TMHMM to predict transmembrane helices. Sequences with zero helices or a single predicted transmembrane helix were retained and considered as secretory proteins. Predicted secreted proteins were compared with those of the Fungal Secretome KnowledgeBase (FunSecKB) database [41] to elucidate their potential function. Furthermore, we used HMMER [42,43] against the Carbohydrate-active enzyme ANnotation database (dbCAN2) [44,45] to search for carbohydrate-active enzymes (CAZymes) in the predicted secreted proteins.

## 3. Results

### 3.1. RNA Reads Processing and Filtering of Fungal Sequences

On average TopHat filtered out 21% of the transcripts from Mangwanda et al. (2015), as they did not map to the *E. grandis* genome (Table 1).

**Figure 1:**
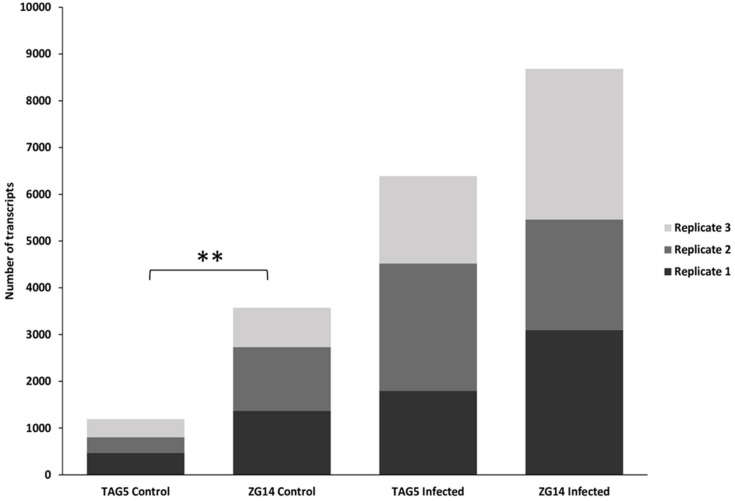
Extracted fungal transcripts between the *E. grandis* clones and treatments. The number of fungal transcripts per replicate extracted from *E. grandis* TAG5 and ZG14 control and infected data sets. ** *p* < 0.01.

In both *E. grandis* clones more fungal transcripts were found in the infected samples compared to the controls (TAG5_Control: 4 ± 1% versus TAG5 _Infected: 24 ± 3%; ZG14_Control: 13 ± 2% versus ZG14_Infected 27 ± 2%). Additionally, control samples of the susceptible clone ZG14 contained a significantly higher number of fungal transcripts (*p* < 0.01) than the resistant clone TAG5 (Figure 1), despite samples of both clones having a similar number of transcripts in total (Table 1). For all samples most transcripts showed good matches with the NCBI protein database (Figure S1). To highlight taxonomic specificity amino acid % identities from blastx were used on one transcript dataset (TAG5_Control2), indicating that our sequences share significantly high sequence similarities with the reference fungal datasets from NCBI (Figure S2).

### 3.2. Taxonomic Affiliation

**Figure 2:**
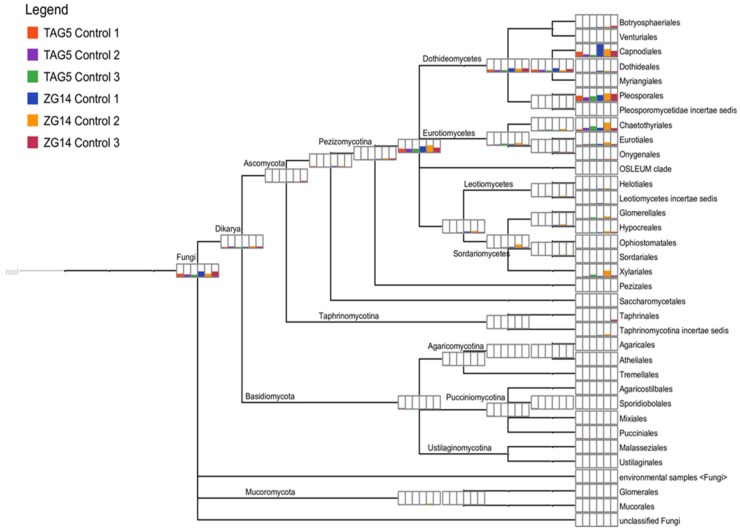
Taxonomic affiliation of extracted fungal transcripts. Taxonomic clustering of fungal transcripts in *E. grandis* TAG5 and ZG14 control samples based on blastx as assigned by MEGAN. The height of the bar indicates the number of transcripts assigned to a node.

The taxonomic affiliations of fungal transcripts revealed a high diversity of fungal taxa (Figure 2). The infection experiment was performed with *C. austroafricana* and the infected samples unsurprisingly contained a large proportion of Sordariomycetes related to this taxon (Figure S3). As we were interested in non-symptomatic fungi, subsequent analyses were only performed on transcripts from control samples. Next to high taxon diversity, we also observed similar taxa between the control TAG5 and ZG14 samples (Figure 2, Figures S5 and S6; Table S4). The majority of the complete set of fungal transcripts (2738) belonged to the *Dothideomycetes*, with the genera *Cercospora* (291), *Sphaerulina* (48), *Ascochyta* (46) and *Epicoccum* (25) dominating (Figure S5). *Pestalotiopsis* was another prevalent genus found, with 140 transcripts amongst all six samples. We also recovered transcripts from Basidiomycota, including *Moniliophthora*, *Fibularhizoctonia*, *Cryptococcus*, *Kondoa*, *Mixia*, *Puccinia*, *Malassezia* and *Moesziomyces* (Figure S5).

### 3.3. GO and KOG Annotation of Transcripts

**Figure 3:**
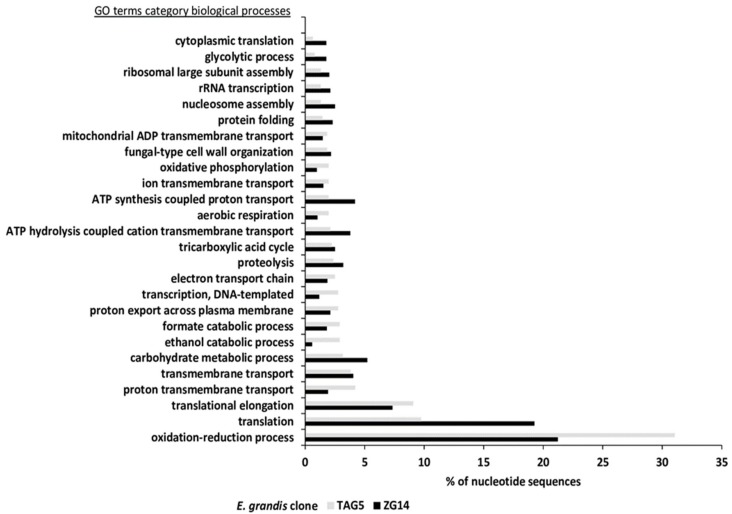
Blast2GO terms in category biological processes. Most abundant gene ontology (GO) terms of fungal transcripts in *E. grandis* clones TAG5 and ZG14 control samples.

Using Blast2GO, 189 biological process terms were inferred in TAG5 and 370 biological process terms in ZG14 (Figure 3 and Figure S7, Table S8). For both data sets transcripts fell mostly into the GO terms ‘oxidation-reduction process’ (TAG5: 31%, ZG14: 21%), ‘translation’ (TAG5: 10%, ZG14: 19%) and ‘translational elongation’ (TAG5: 9%, ZG14: 7%). Fungal transcripts identified as ‘ent-kaurene oxidase’ were only found in the TAG5 and not in ZG14 samples (Tables S9 and S10).

After using GenMarkS-T to translate nucleotide into protein sequences, we aligned all fungal sequences against functional databases to help interpret the expressed fungal genes (Table 2). In all categories we found more proteins in the ZG14 control samples compared to the TAG5 control samples.

**Figure 4:**
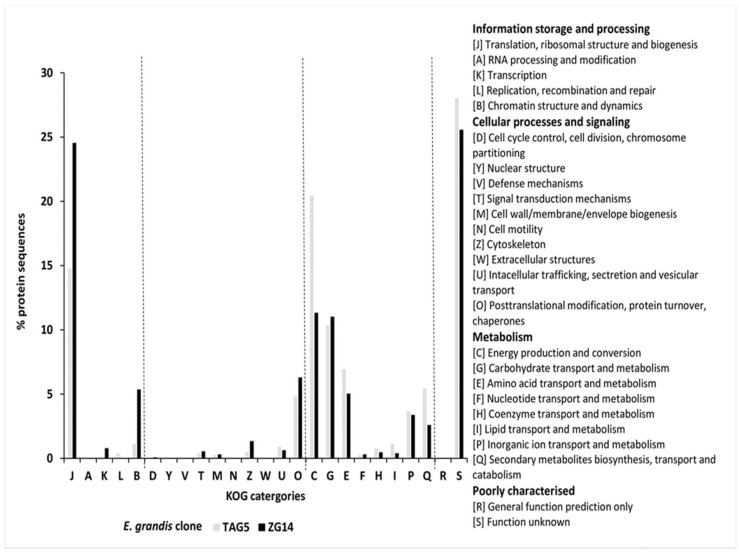
KOG classification of fungal transcripts extracted for *E. grandis* TAG5 and ZG14 control data sets.

Out of all translated fungal proteins, 75% of the TAG5 data and 90% of the ZG14 data aligned to the KOG database. Functional annotation of fungal transcripts covered most of the known KOG categories (Figure 4 and Table S11), whereby the majority of transcripts belonged to general housekeeping cellular functions associated with translation (J), energy production (C) and carbohydrate transport and metabolism (G). Transcripts were additionally predicted in classes potentially involved in fungal–plant interactions, i.e., inorganic ion transport and metabolism (P), secondary metabolite biosynthesis, transport and catabolism (Q) and a few transcripts in intracellular trafficking, secretion, and vesicular transport (U). Furthermore, some transcript categories tended to be different between the *E. grandis* clones. TAG5 control samples showed more protein sequences in the metabolism categories C, E, P and Q, whereas ZG14 control samples had more protein sequences in the group information storage and processing, i.e., J, K and B.

### 3.4. Identification of Proteins Involved in Pathogen–Host Interaction

**Figure 5:**
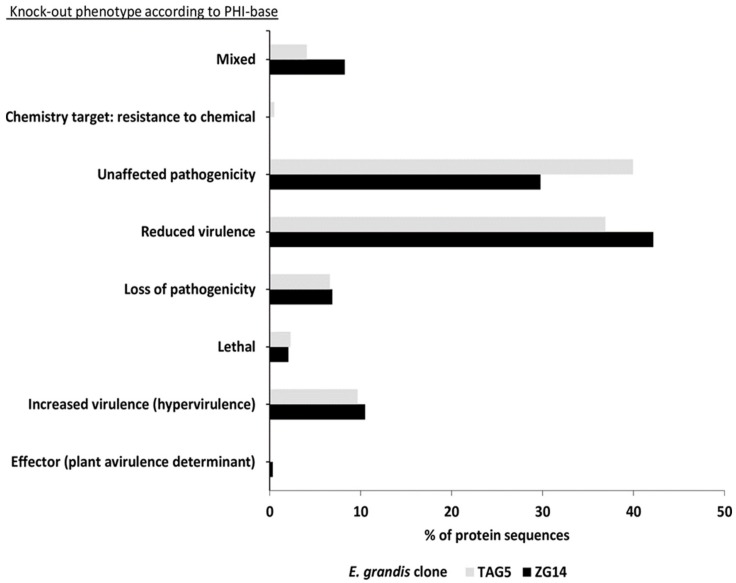
PHI-base analysis for TAG5 and ZG14 control data sets. Indicated are the number of transcripts in the individual phenotype categories.

Out of all fungal protein sequences aligned to the PHI-base, 37% of the TAG5 data and 41% of the ZG14 data resulted in information on genes that affect the outcome of pathogen–host interactions (Figure 5, Table S12). Thereby, the fungal proteins from the two *E. grandis* clones showed a similar distribution of PHI-base knockout phenotypes. Of 393 transcripts with a PHI-base hit, 236 showed an effect on pathogenicity for TAG5, whereas 157 had no known effect. Of 581 transcripts with a PHI-base hit in the ZG14 data, 408 showed an effect on pathogenicity and 173 had no known effect. Most proteins found affecting pathogenicity fell into the category “reduced virulence” (TAG5: 37%, ZG14: 42%), whereas 10% of the proteins showed increased virulence compared to the wild-type. An additional 2% of the fungal proteins were potentially essential for pathogen survival as knockouts of their homologs result in a lethal phenotype.

### 3.5. Secreted Proteins

**Figure 6:**
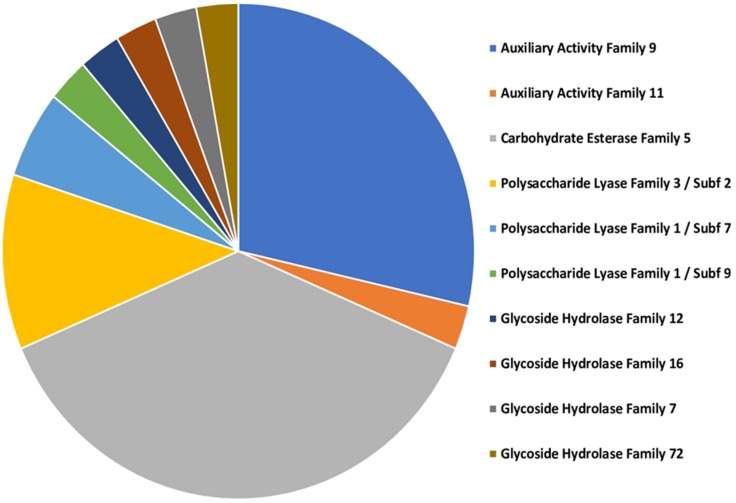
Carbohydrate-active enzymes (CAZymes) of predicted secreted protein for TAG5 and ZG14 control data sets combined.

Using the FunSecKB database we found 45 predicted secreted proteins for TAG5 and 96 for ZG14 transcripts (Table 2, Tables S13–S15). The majority of the proteins aligned against the dbCAN2 database fell into carbohydrate esterase family 5 (13) and auxiliary activity family 9 (10) (Figure 6, Table S16). Combined, 13 proteins belonged to the carbohydrate esterase, 11 to the auxiliary activity, seven to the polysaccharide lyase and four to the glycoside hydrolase CAZyme family.

## 4. Discussion

In this study, we successfully characterised fungal reads from transcriptome data sets of stem tissue of *E. grandis* to understand fungal gene expression in a resistant and in a susceptible eucalypt clone.

### 4.1. Eucalyptus Fungal Community Includes Known Plant Pathogenic Taxa

Sequences associated with various fungal taxa from different evolutionary lineages were found in the *Eucalyptus* stem tissue. Our results indicate that the fungal community is strongly dominated by *Ascomycota*, which confirms metabarcoding studies on the phyllosphere of *Eucalyptus* [9,46]. *Dothideomycetes* were by far the most abundant class in healthy stem tissue, irrespective of the trees belonging to the resistant or the susceptible clone. This is not unexpected, as it confirms most studies using a metabarcoding approach, but also a study comparing RNA-seq data of various *Picea abies* tissues [20]. RNA-seq data has been used for taxonomic identification in other systems (e.g., [47]), but there is still a lack of established reference genomes and bioinformatic pipelines, especially in fungal–plant data sets. Taxonomic inferences may also be limited by the depth of RNA-seq, as host transcripts are usually highly abundant.

*Dothideomycetes* contain a large amount of plant pathogenic fungi and we found several fungal taxa that are potentially pathogenic on *Eucalyptus*, again confirming other studies using metabarcoding sequencing [9]. Members of the *Capnodiales* and the *Pleosporales* were prevalent in our study and these orders are known to harbor many *Eucalyptus* pathogens, including those in the *Didymellaceae*, *Mycosphaerellaceae* and *Teratosphaeriaceae* families. However, compared to amplicon-based studies, which are unable to differentiate between living and dead material, our study using RNAseq data indicates that these taxa are actually metabolically active members of the microbial community. Substantiating an active role of these fungi is an isolation study that also recovered many of these taxa as endophytes from *Eucalyptus* [48]. Taken together all these studies indicate that healthy tissue of *E. grandis* contains large proportions of potentially pathogenic taxa, which live without causing disease symptoms, but are metabolically active in the plants.

### 4.2. Resistance Breeding Influences the Activity of the Associated Fungal Community 

One of the most outstanding results of our study was the differential abundance of fungal transcripts between clones. Control samples of the resistant *E. grandis* clone TAG5 had a significantly lower abundance in fungal transcripts and therefore most likely a lower fungal biomass than the susceptible clone ZG14, despite both having a similar amount of overall sequencing reads. There is indeed increasing evidence that plant response to pathogens has effects on other fungi associated with the plant. Needles of *Pinus radiata* susceptible to *Cyclaneusma* needle-cast for instance, contained more culturable fungi than trees that did not show any symptoms [49]. Similarly, the xylem of individuals of Dutch elm disease-susceptible *Ulmus minor* clones harbored a greater diversity and higher density of endophytic fungi than resistant *U. minor* and *Ulmus pumila* clones [50]. Especially the latter study also indicates that genetic factors related to pathogen resistance are important in influencing the structure of fungal communities. This is substantiated by a genome-wide association study (GWAS) that identified loci correlated with the structure of microbial communities in *Arabidopsis thaliana*. A large proportion of these loci are known to be involved in plant defence [51].

In addition to the transcript abundance we could show that fungal diversity recovered from a resistant and susceptible *E. grandis* genotype was similar, but the transcript abundance and thereby their activity was strongly influenced by host genotype. It has been proposed before that plant domestication and breeding against pathogens has most likely unintendedly also led to selection against other members of diverse plant-associated microbial communities [52]. Although further studies are needed to substantiate the specific outcomes of such unintentional breeding properties, our study demonstrates strong effects of the plant genotype on the fungal community. These should be considered in tree breeding, as beneficial members of fungal communities will also be affected.

Functionally the fungal communities of TAG5 and ZG14 were similar, but there were some notable differences. The community of the resistant TAG5 clone showed more activity in secondary metabolite biosynthesis, transport and catabolism, amino acid transport and metabolism and energy production and conversion, whereas the fungal community of ZG14 was more active in translation, ribosomal structure and biogenesis, transcription and chromatin structure and dynamics. The environment in a susceptible *E. grandis* clone could be more conducive for building fungal cellular structures and biomass growth, whereas fungi in the resistant clone invest more in secondary metabolism as a response to the stronger host defences.

### 4.3. The Potential Function of Genes Transcribed by the Fungal Community in Non-Symptomatic Stem Tissue

#### 4.3.1. Fungal Virulence and Host Interaction

Most of the predicted fungal proteins in our study that had a match in the PHI-base showed knock-out phenotypes for reduced virulence, followed by unaffected pathogenicity. In cases where individuals of the fungal community penetrate their host they need to overcome host defence by employing a range of effectors to modulate host cellular functions and immune responses [53]. As we found many similar predicted proteins as those found in pathogenic fungi, our results indicate that asymptomatic fungi use the same molecular mechanisms as pathogenic fungi to counter existing plant defences. If this can be confirmed in future studies, it would also be a likely explanation on why the activity of the fungal community in the resistant clone TAG5 is reduced compared to the susceptible trees. The same plant factors that hinder *C. austroafricana* from becoming pathogenic also compromise the interaction between resistant trees and other fungi.

In our study we found the highest number of fungal transcripts belonging to the GO class ‘oxidation-reduction process’ and furthermore several ‘oxidoreductase’ hits in the predicted secreted proteins. Although most of these processes will be involved in intracellular processes, fungi are also utilizing reactive oxygen species (ROS) for extracellular purposes as signal molecules, for the extracellular degradation of lignocellulose and in the interaction with their plant hosts and other microbes [54]. ROS are used by fungal cells to decide between growth and cell differentiation [55]. Secreted oxygen radicals are also important in the degradation of the highly resistant polymeric structure of lignocellulose and are possibly used as a carbon source for fungal growth (discussed below). We found more fungal transcripts for oxidation-reduction processes for the TAG5 samples, which could be an indication that those fungi are challenged with a stronger plant ROS defence than the fungi in ZG14.

We also found hints that some fungi can interfere with growth hormones of their host, as we detected transcripts for ‘ent-kaurine oxidase’, an enzyme that catalyses the biosynthesis of gibberellic acid (GA) expressed in TAG5 samples. Mangwanda et al. (2015) found a decrease in plant GA expression in the same *E. grandis* TAG5 samples, which could be related to our findings but requires further work [56].

#### 4.3.2. Plant Penetration and Fungal Nutrition through Polysaccharide Degradation

The fungal transcripts in our study contained several carbohydrate-active enzyme (CAZyme) families, including carbohydrate esterases (CEs), glycoside hydrolases (GHs), polysaccharide lyases (PLs), and auxiliary activities (AA). Plant cell wall carbohydrates, in the form of cellulose, hemicelluloses, pectin, and lignin, make up the major part of stems and provide physical stability, but also function as a physical barrier to plant pathogens. However, fungi have evolved ways to degrade these carbohydrates in order to successfully colonise stem tissues and some even use them as a carbon source [57]. Biotrophic fungi, including many plant pathogens, often modify or disrupt cell walls by using cell wall-degrading enzymes, which form part of a large family of CAZymes.

Cutinases, which belong to the CE family, were abundantly found in the predicted secreted proteins of this study. Although nothing is known about their function in non-symptomatic plant–fungus interactions, they are known to play important roles in pathogenic fungi. They facilitate fungal penetration through the plant cuticle by the hydrolysis of ester bonds from the fatty acid polymers in cutin [58]. Additionally, cutinases are important for fungal spore attachment to the host [59,60], as well as surface signaling between plant and fungi [61].

CAZymes of the AA9 family were the second largest group found in the predicted secreted proteins of our study. AA9 lytic polysaccharide monooxygenases catalyse the cleavage of glycosidic bonds in cellulose and some also lyse various hemicellulosic substrates [62]. Again, nothing is known about their function in non-symptomatic interactions, but the CAZymes in this family are known to be important in plant invasion and virulence of fungal pathogens. Genome comparison of *Zymoseptoria tritici* (syn. *Mycosphaerella graminicola*) to the more aggressive pathogen *Phaeosphaeria nodorum* revealed that the former only contained two AA9 genes, whereas 30 AA9 genes could be found in *P. nodorum* [63]. AA9 CAZymes are also involved in biodegradation of wood, as the genome of the white-rot fungus and phytopathogen *Heterobasidion irregulare* encodes an abundance of putative AA9 genes which reveals its ability for the degradation of cellulose and hemicellulose [64].

The analysed fungal community of *E. grandis* has the potential to degrade complex polysaccharides, as they contain many GH enzymes, which modify the polysaccharide backbones of cellulose and hemicelluloses [65], as well as PL enzymes, which degrade pectin backbones [66]. Although the analysed samples were visually not infected, it is tempting to speculate that the fungi of these communities also use CAZymes for the penetration of the plants cell surface and potentially use the degraded tissue as a carbon source. We found high numbers of carbohydrate metabolism transcripts in the KOG classification ([G] Carbohydrate transport and metabolism) as well as in the blast2GO analyses (Carbohydrate metabolic process), which might indicate the additional utilisation of carbon as a food source through degrading the plant cell wall.

## 5. Conclusions

Functional studies on plant-associated fungal communities are encumbered by the overwhelming majority of transcripts belonging to the host plant and not the fungi in the community. By extracting fungal transcripts from two *E. grandis* wood mRNA datasets we were able to describe fungal transcripts taxonomically and functionally to a level that enabled the discovery of three interesting findings that validate further study. Firstly, transcripts from fungal communities between susceptible and resistant clones consisted of similar taxa, indicating that resistance breeding does not necessarily affect the diversity of active members in the fungal tree community. Secondly, transcribed genes in the fungal communities were similar between the two *E. grandis* clones, with notable differences in secondary metabolism and biomass growth. Thirdly, the number of fungal transcripts was lower in the resistant *E. grandis* clone compared to the susceptible one, adding evidence that the host genotype is a strong determinant of fungal metabolic activity.

Our study shows that fungi in non-symptomatic interactions communicate in a very similar way with their plant host as both mutualistic and pathogenic fungi. They secrete proteins for interfering with plant communication via effectors and oxireductases and digest plant material to potentially penetrate plants using CAZymes. Many of the active taxa that we inferred from our dataset are potential pathogens that increase activity when their host is exposed to other biotic or abiotic stresses. Therefore, in order to guide future efforts in breeding plantation tree and crop varieties to increase plant performance, it is vital to understand how these non-symptomatic but intimate relationships between plants and microbes are initiated, established and controlled.

## Figures and Tables

**Table 1:** Overview of the processed nucleotide sequences. Shown are the number of reads, or transcripts for each sample. Percentages were calculated with the number of the previous filtering step.

	Raw Reads (Forward; Reverse)	Unmapped Transcripts after TopHat	Unmapped %	Transcripts after Trinity	Trinity %	Fungal Transcripts	Fungi %
TAG5_Control_BR1TP1	39,273,762; 39,273,762	8,093,926	20.61	8689	0.11	472	5.43
TAG5_Control_BR2TP1	39,195,029; 39,195,029	8,012,422	20.44	8934	0.11	335	3.75
TAG5_Control_BR3TP1	39,370,778; 39,370,778	8,152,959	20.71	9111	0.11	383	4.20
ZG14_Control_ BR1TP1	37,594,917; 37,594,917	8,052,598	21.42	9231	0.11	1373	14.87
ZG14_Control_ BR2TP1	38,697,190; 38,697,190	8,483,221	21.92	9138	0.11	1358	14.86
ZG14_Control_ BR3TP1	38,856,446; 38,856,446	7,875,385	20.27	8922	0.11	843	9.45
TAG5_Infected_BR1TP1	37,617,103; 37,617,103	7,323,565	19.47	6860	0.09	1798	26.21
TAG5_Infected_BR2TP1	37,390,551; 37,390,551	7,952,651	21.27	10,526	0.13	2722	25.86
TAG5_Infected_BR3TP1	38,684,116; 38,684,116	8,488,972	21.94	9973	0.12	1870	18.75
ZG14_Infected_ BR1TP1	38,062,937; 38,062,937	7,919,759	20.81	10,687	0.13	3096	28.97
ZG14_Infected_ BR2TP1	34,373,634; 34,373,634	7,177,341	20.88	9888	0.14	2364	23.91
ZG14_Infected_ BR3TP1	36,626,103; 36,626,103	7,778,486	21.24	10,954	0.14	3221	29.40

**Table 2:** Overview of the processed protein sequences. Shown are the numbers of functional proteins of TAG5 and ZG14 control samples that aligned against respective databases.

	EnTAP/GenMarkS-T	PHI-base	KOG	SignalP	TargetP	TMHMM	FunSecKB	dbCAN2
TAG5 Control	1061	396	793	108	103	79	45	11
ZG14 Control	1405	581	1271	153	142	121	96	24

EnTAP: Eukaryotic Non-Model Transcriptome Annotation Pipeline, PHI: Pathogen Host Interaction database, KOG: EuKaryotic Orthologous Groups, SignalP: predicts secreted proteins, TargetP: filters out mitochondrial proteins, TMHMM: predicts transmembrane helices, FunSecKB: Fungal Secretome KnowledgeBase, dbCAN2: Carbohydrate-active enzyme ANnotation database.

## Supplementary Materials

The following are available online at https://www.mdpi.com/2076-2607/7/10/375/s1, Figure S1: Distribution of e-values for assigned transcripts against the NCBI protein database for each sample, Figure S2: TAG 2 Control specificity to NCBI nr database, Figure S3: MEGAN graph TAG5 and ZG14 infected samples, Table S4: Assigned transcript numbers to taxa node in MEGAN tree (Figure 2), Figure S5: Assigned transcripts numbers to taxa nodes class, order and genus based on blastx assigned by MEGAN, Figure S6: PCoA TAG5 and ZG14 control samples, Figure S7: Blast2GO results all hits as graphs, Table S8: Biological processes blast2GO results TAG5 and ZG14, Table S9: Biological processes blast2GO TAG5, only ‘oxidation-reduction process’ result, Table S10: Biological processes blast2GO ZG14, only ‘oxidation-reduction process’ result, Table S11: KOG classification through eggNOG output using taxonomic scope “fungi” (fuNOG) and DIAMOND blastp, Table S12: PHI-base output for TAG5 and ZG14 control data sets against PHI 4.5 database using DIAMOND blastp, Table S13: Predicted secreted proteins TAG control data sets, blastp output, Table S14: Predicted secreted proteins ZG14 control data sets, blastp output, Table S15: Only hits for predicted secreted proteins for TAG5 and ZG14 control data sets, Table S16: Predicted secreted CAZymes HMMER against dbCAN2.
